# Supplementary figures and images for: Genetic targeting of Card19 is linked to disrupted NINJ1 expression, impaired cell lysis, and increased susceptibility to Yersinia infection
Source: PLoS Pathog. 2021 Oct 14;17(10):e1009967. doi: 10.1371/journal.ppat.1009967 (PMC8547626; doi:10.1371/journal.ppat.1009967)

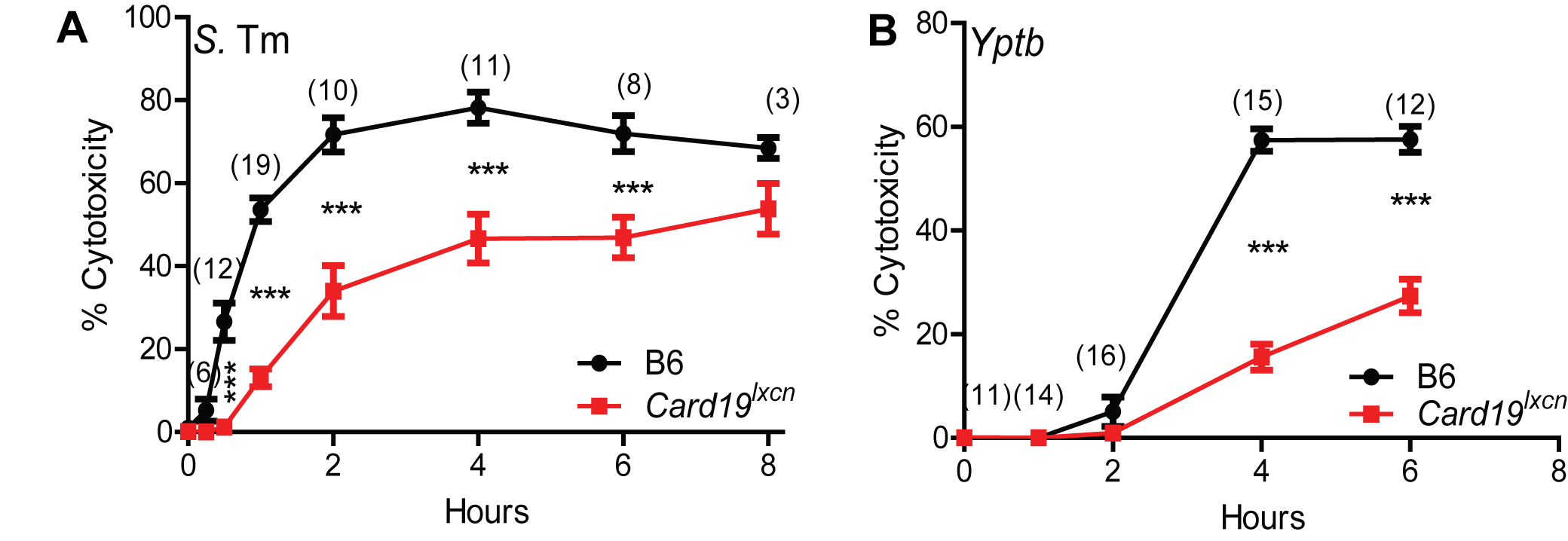

Supplement: S1 Fig — Primary C57BL/6J (B6), Card19-/-, Card19+/+, and Casp11-/- BMDMs were treated with (A) S. Tm (B) Yptb, and cell death was assayed by LDH release. BMDMs were treated, supernatants were harvested from triplicate wells at indicated time points and measured for cytotoxicity. The mean ± SEM of means from triplicate wells from 3–19 independent experiments as indicated in parenthesis. *** p < 0.001, ** p < 0.01, * p < 0.05. n.s. not significant. 2-way ANOVA with Bonferroni multiple comparisons post-test. (TIF) [file ppat.1009967.s001.tif]

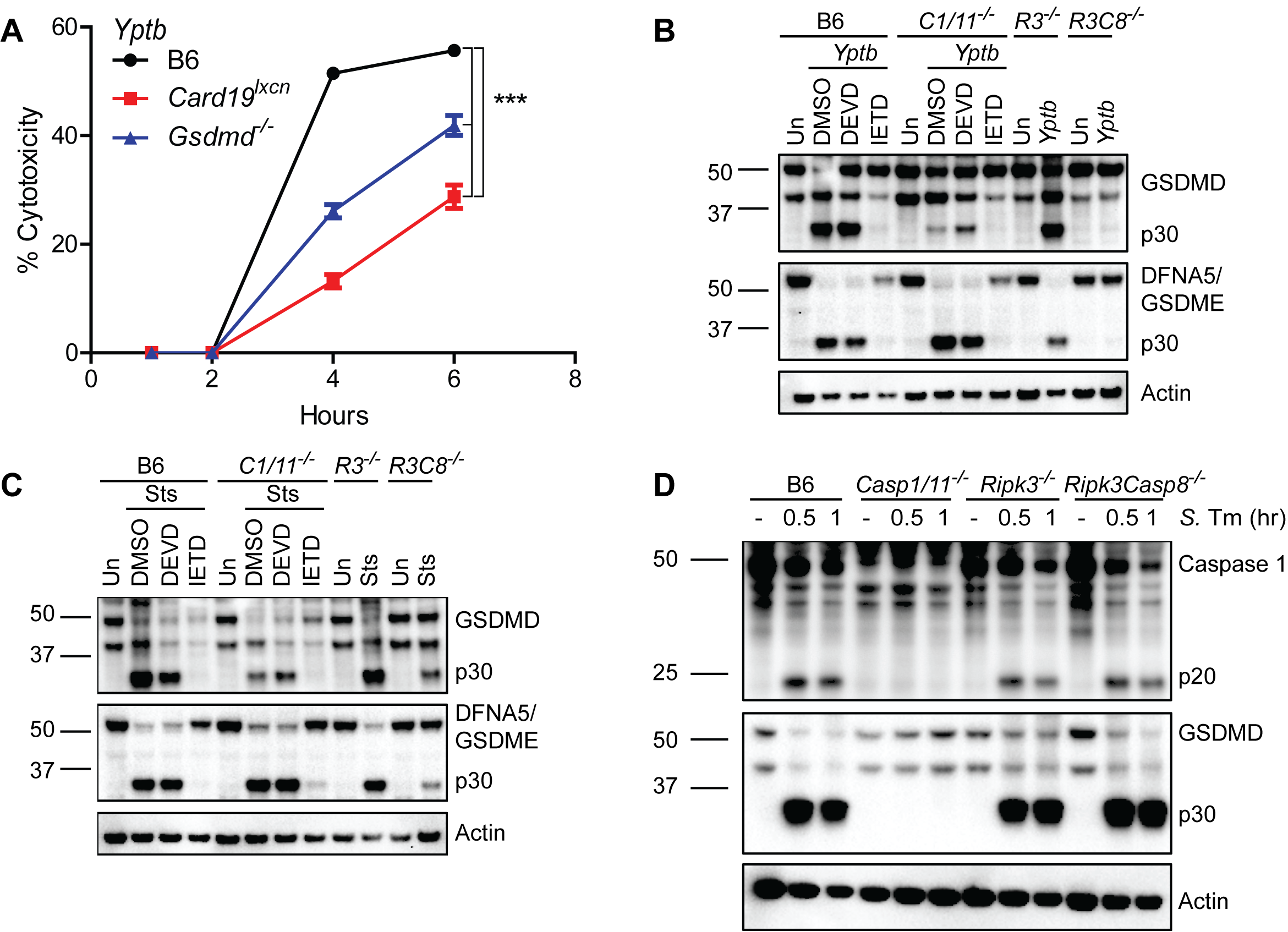

Supplement: S2 Fig — (A) B6, Card19lxcn, and Gsdmd-/- BMDMS were infected with Yptb. Cell death was assayed by LDH release. Representative of three independent experiments. (B-D) B6, Casp1/11-/-, Ripk3-/-, or Ripk3-/-/Casp8-/- BMDMs were left uninfected (Un) (B) infected with Yptb, (C) treated with sts or (D) infected with S. Tm in the presence of DMSO, the caspase-3/7 inhibitor DEVD, and the caspase-8 inhibitor IETD. Lysates were harvested (B, C) 3 hours or (D) 0.5 and 1 hour post treatment and analyzed by western blotting for GSDMD, DFNA5/GSDME, Caspase-1, and actin (loading control). Blots representative of two or three independent experiments. (TIF) [file ppat.1009967.s002.tif]

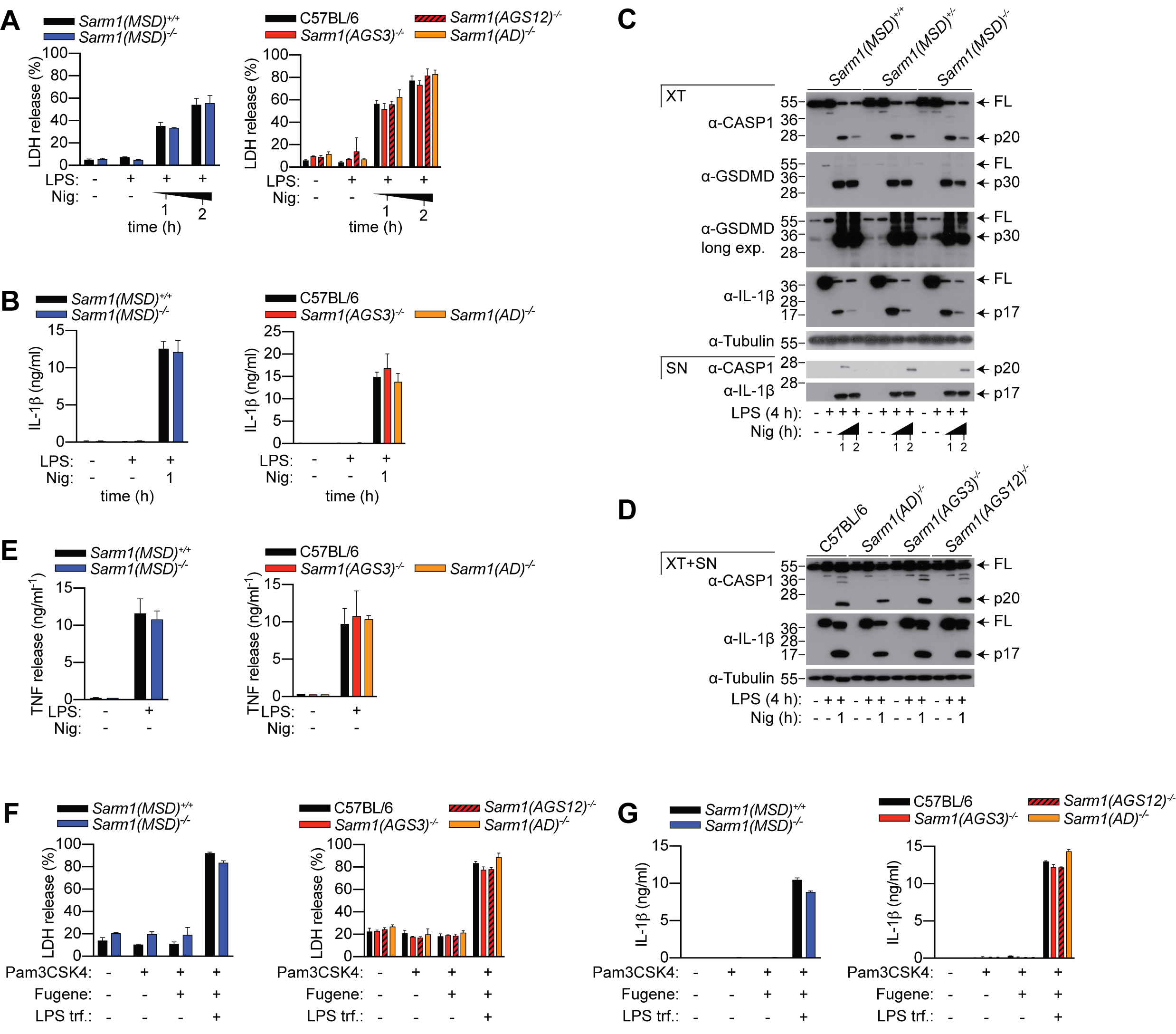

Supplement: S3 Fig — (A-D) Sarm1(MSD)-/-, Sarm1(MSD)+/-, Sarm1(MSD)+/+, C57BL/6, Sarm1(AGS3)-/-, Sarm1(AGS12)-/- and Sarm1(AD)-/- BMDMs were primed with LPS (100 ng/ml) for 4 hours and stimulated with nigericin (5 μM). (A) LDH and (B) IL-1β release were measured at the indicated time points. (C) Supernatant and cell extract or (D) mixed supernatant and cell extract were examined by immunoblotting at the indicated time points. (E) BMDMs were primed with LPS (100 ng/ml) and TNF release was measured after 4 hours. (F and G) BMDMs were primed with Pam3CSK4 (1 μg/ml) for 4 h and were transfected with 2 μg/ml E. coli O111:B4 LPS with Fugene HD. (F) LDH and (G) IL-1β release were measured after 16 h. (A and B, E-G) Data are mean + SD of triplicate cell stimulation and is representative of two to four independent experiments. Immunoblots are representative of two independent experiments. (TIF) [file ppat.1009967.s003.tif]

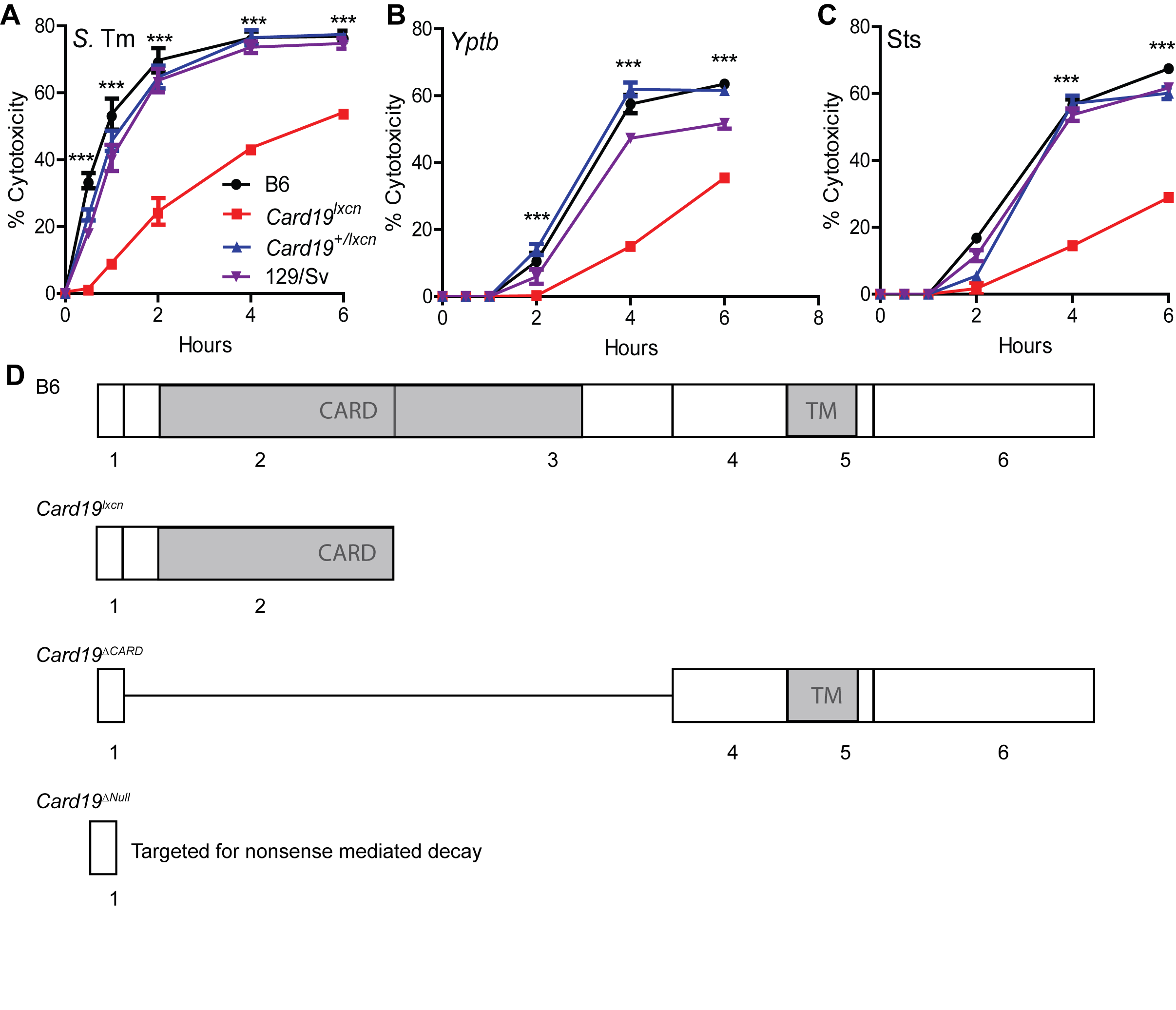

Supplement: S4 Fig — (A-C) B6, Card19lxcn, Card19+/lxcn, and 129/Sv BMDMs were treated with (A) S.Tm, (B) Yptb or (C) sts. Cell death was assayed by LDH release at indicated time points. Representative of three independent experiments. (D) CARD19 exon schematic showing each independent CARD19 mouse line with the respective CARD19 product. (TIF) [file ppat.1009967.s004.tif]

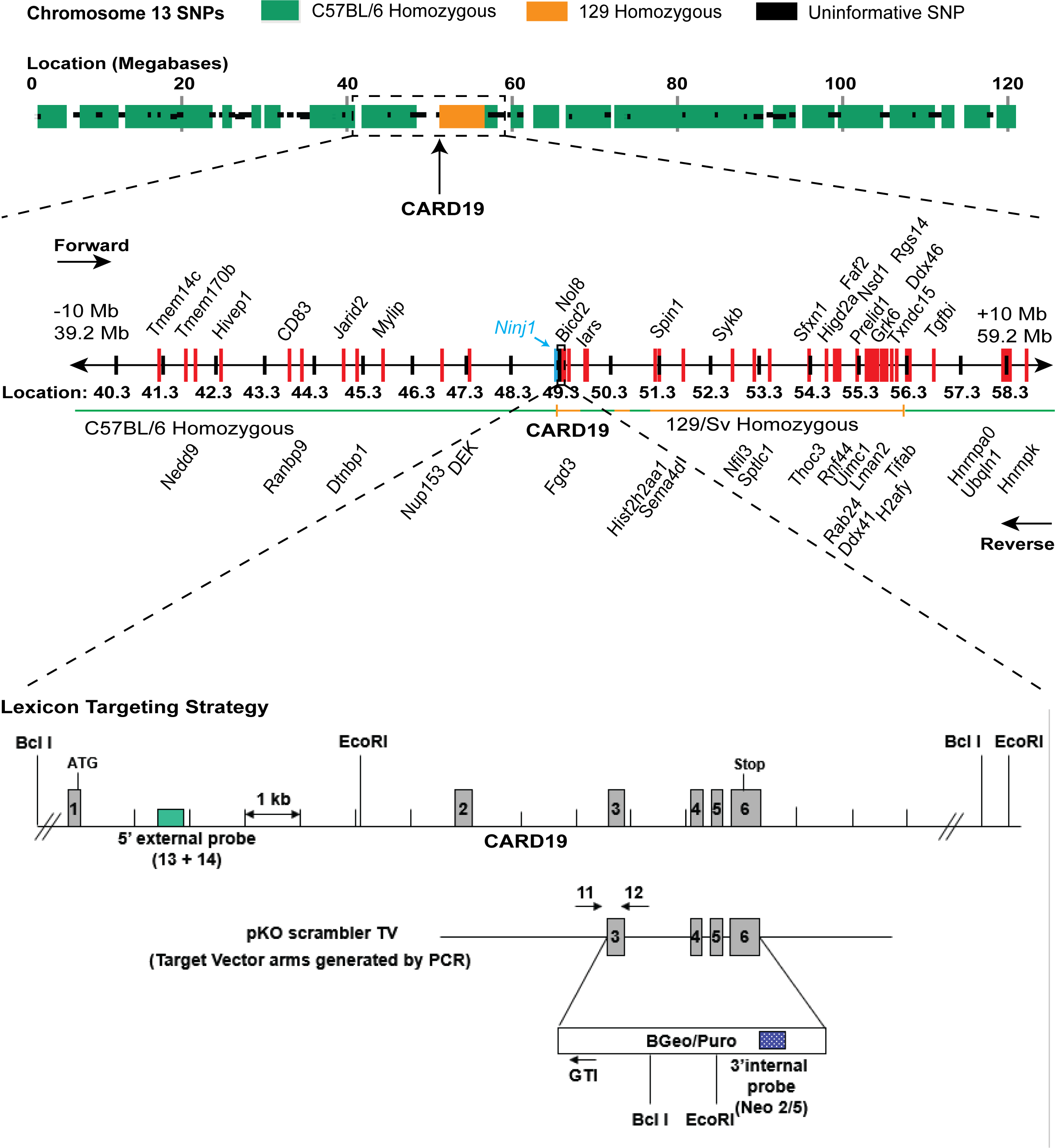

Supplement: S5 Fig — (A) Chromosome 13 with tested SNPs from DartMouse genetic background check. C57BL/6 SNPs are in green, 129SvEvBrd SNPs are in yellow, and uninformative SNPs (i.e. not all samples gave identical results) are in black. The 10 megabase region on either side of the Card19 locus is zoomed in below it with chromosomal locations noted in bold numbers and black notches. Genes in red are expressed in macrophages. Ninj1 is in blue. The six megabase region highlighted by the yellow bar is homozygous for 129SvEvBrd. The green regions are homozygous for C57BL/6. A zoomed in region at Card19 is highlighted with the original Lexicon Targeting strategy. (B) Wildtype, Card19lxcn, and Ninj1-/- iBMDMs were reconstituted with NINJ1/BH1.11 piggyBac or empty vector. Lysates were harvested and run on SDS-PAGE gel and probed for NINJ1 and actin (loading control). (TIF) [file ppat.1009967.s005.tif]

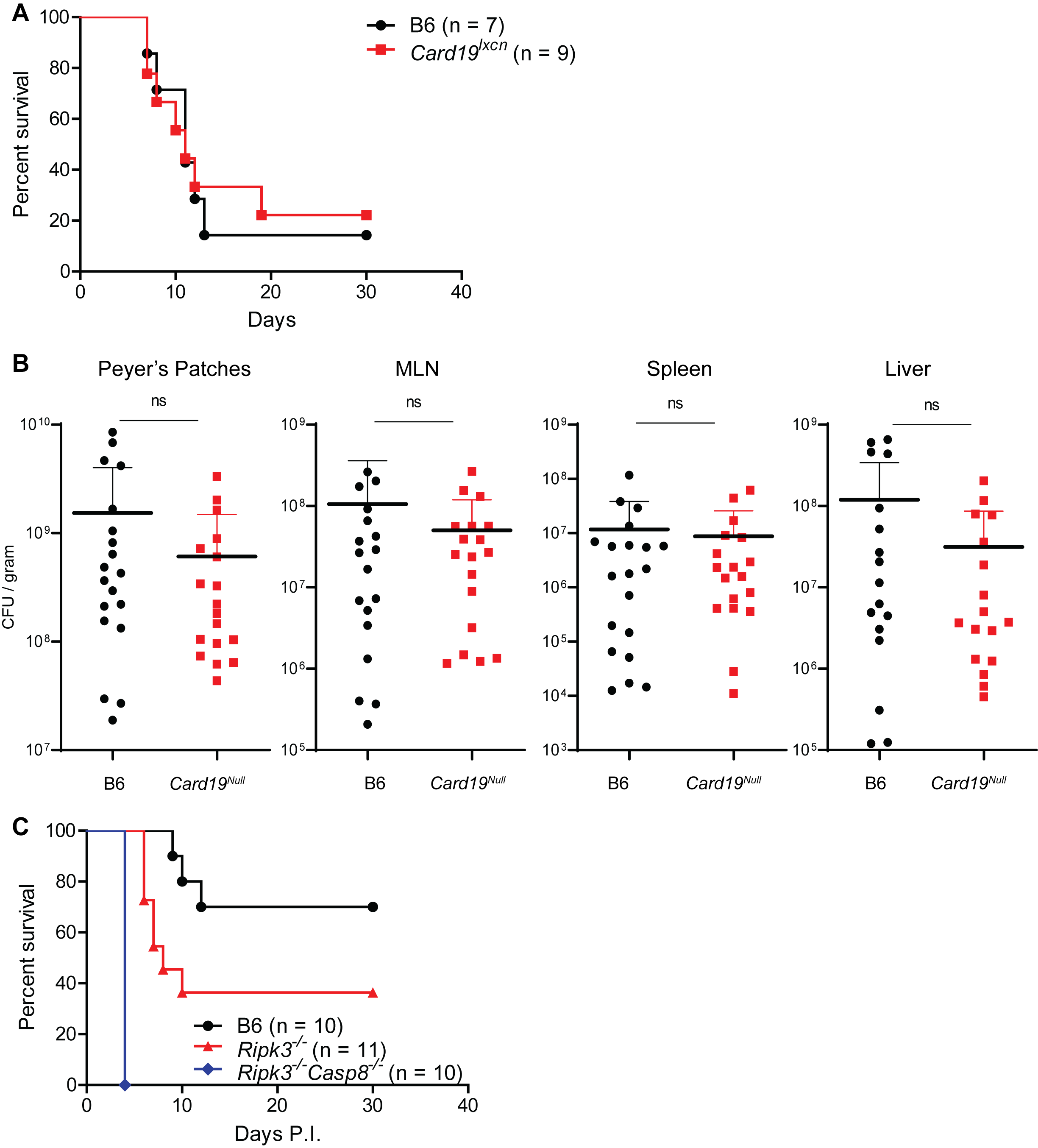

Supplement: S6 Fig — (A) Survival of B6 and Card19lxcn mice following oral infection 107 CFUs of strain SL1344 S. Tm. Data are from one experiment. (B) B6 and Card19Null mice were orally infected with 108 CFUs of strain IP2777 Yptb. Seven days post infection, organs were harvested for enumeration. Data pooled from three independent experiments (B6 = 20, Card19Null = 17). (C) Survival of B6, Ripk3-/-, and Ripk3-/-Casp8-/- mice following oral infection 108 CFUs of strain IP2777 Yptb. Data are pooled from two independent experiments. (TIF) [file ppat.1009967.s006.tif]
