# Supplementary material for: Genetic targeting of Card19 is linked to disrupted NINJ1 expression, impaired cell lysis, and increased susceptibility to Yersinia infection
Source: PLoS Pathog. 2021 Oct 14;17(10):e1009967. doi: 10.1371/journal.ppat.1009967 (PMC8547626; doi:10.1371/journal.ppat.1009967)
Supplement: S1 Table — Sources of BMDMs and their derivation are listed in S1 Table. (DOCX) [file ppat.1009967.s007.docx]

**S1 Table: BMDM and Murine Sources**

| **Macrophages** | **Mice Generation** | **References** |
| --- | --- | --- |
| Card19^lxcn^ | 129 ESC and backcrossed to B6 | Rios et al. 2020 |
| Card19^ΔCARD^ | C57BL/6J CRISPR Line | This paper |
| Card19^null^ | C57BL/6J CRISPR Line | This paper |
| Sarm1(MSD)^-/-^ | 129 ESC and backcrossed to B6 | Szrette et al. 2009 |
| Sarm1(AD)^-/-^ | 129 ESC and backcrossed to B6 | Kim et al., 2007, JAX stock #018069 |
| Sarm1(AGS3)^-/-^ | C57BL/6J CRISPR Line | Uccellini et al., 2020, JAX stock #034399 |
| Sarm1(AGS12)^-/-^ | C57BL/6J CRISPR Line | Uccellini et al., 2020 |

Sources of BMDMs and their derivation are listed in S1 Table.
