# Supplementary material for: Genetic targeting of Card19 is linked to disrupted NINJ1 expression, impaired cell lysis, and increased susceptibility to Yersinia infection
Source: PLoS Pathog. 2021 Oct 14;17(10):e1009967. doi: 10.1371/journal.ppat.1009967 (PMC8547626; doi:10.1371/journal.ppat.1009967)
Supplement: S2 Table — SNPs from DartMouse SNP genetic background check 10 megabases upstream and downstream of the Ninj1/Card19 chromosome 13 locus. SNP identification and chromosomal position are indicated. SNPs are indicated as wildtype (B6) or 129SvEvBrd (129). The change, gene ID, gene name, and known function are listed. Ninj1 and Card19 are listed for reference. (DOCX) [file ppat.1009967.s008.docx]

**S2 Table: Chromosome 13 SNP Results**

| SNP | Location | B6/129 | Change | Gene ID (ENMUSG) | Gene Name | Function |
| --- | --- | --- | --- | --- | --- | --- |
| rs30100204 | 39337735 | B6 | intergenic variant |  |  |  |
| rs36514425 | 39958176 | B6 | intergenic variant |  |  |  |
| rs29228586 | 40345334 | B6 | intron variant | [00000047094](https://uswest.ensembl.org/Mus_musculus/Gene/Variation_Gene/Table?db=core;g=ENSMUSG00000047094;source=dbSNP;v=rs29228586;vdb=variation;vf=12743877) | orofacial cleft 1 candidate | associated with cleft lip |
| rs50931018 | 42450412 | B6 | intergenic variant |  |  |  |
| rs29236582 | 42602578 | B6 | intergenic variant |  |  |  |
| rs29635560 | 42602754 | B6 | intergenic variant |  |  |  |
| rs13481783 | 42840147 | B6 | intron variant | [00000054728](https://uswest.ensembl.org/Mus_musculus/Gene/Variation_Gene/Table?db=core;g=ENSMUSG00000054728;source=dbSNP;v=rs13481783;vdb=variation;vf=12824581) | phosphatase and actin regulator 1 | motility and cytoskeletal organization |
| rs46355744 | 43000858 | B6 | intron variant | [00000054728](https://uswest.ensembl.org/Mus_musculus/Gene/Variation_Gene/Table?db=core;g=ENSMUSG00000054728;source=dbSNP;v=rs46355744;vdb=variation;vf=12830223) |  |  |
| rs29552103 | 43005082 | B6 | intron variant | [00000054728](https://uswest.ensembl.org/Mus_musculus/Gene/Variation_Gene/Table?db=core;g=ENSMUSG00000054728;source=dbSNP;v=rs29552103;vdb=variation;vf=12830382) |  |  |
| rs51682551 | 43032240 | B6 | intron variant | [00000054728](https://uswest.ensembl.org/Mus_musculus/Gene/Variation_Gene/Table?db=core;g=ENSMUSG00000054728;source=dbSNP;v=rs51682551;vdb=variation;vf=12831442) |  |  |
| rs3712907 | 43171011 | B6 | intron variant | [00000021368](https://uswest.ensembl.org/Mus_musculus/Gene/Variation_Gene/Table?db=core;g=ENSMUSG00000021368;source=dbSNP;v=rs3712907;vdb=variation;vf=12836787) | Tbc1d7 | cell growth and differentiation |
| rs29995243 | 43206944 | B6 | intron variant | [00000051335](https://uswest.ensembl.org/Mus_musculus/Gene/Variation_Gene/Table?db=core;g=ENSMUSG00000051335;source=dbSNP;v=rs29995243;vdb=variation;vf=12838252) | glucose-fructose oxidoreductase domain containing 1 | |
| rs29864465 | 44039283 | B6 | intergenic variant |  |  |  |
| rs46963560 | 45010065 | B6 | intergenic variant |  |  |  |
| rs6296954 | 45351934 | B6 | intergenic variant |  |  |  |
| rs3688207 | 45359288 | B6 | intergenic variant |  |  |  |
| rs108216631 | 45395035 | B6 | intron variant | [00000038175](https://uswest.ensembl.org/Mus_musculus/Gene/Variation_Gene/Table?db=core;g=ENSMUSG00000038175;source=dbSNP;v=rs108216631;vdb=variation;vf=12912010) | Idol/Mir/Myelip | lipid metabolism |
| rs29225851 | 46060929 | B6 | intron variant | [00000221550](https://uswest.ensembl.org/Mus_musculus/Transcript/Summary?db=core;g=ENSMUSG00000113860;r=13:46214073-46222241;t=ENSMUST00000221550.2;v=rs29225851;vdb=variation;vf=12933971) | predicted gene |  |
| rs3663819 | 46297108 | B6 | intron variant | [00000063529](https://uswest.ensembl.org/Mus_musculus/Gene/Variation_Gene/Table?db=core;g=ENSMUSG00000063529;source=dbSNP;v=rs3663819;vdb=variation;vf=12942426) | stathmin domain containing 1 | cell differentiation |
| rs6411274 | 47129920 | B6 | intron variant | [00000038068](https://uswest.ensembl.org/Mus_musculus/Gene/Variation_Gene/Table?db=core;g=ENSMUSG00000038068;source=dbSNP;v=rs6411274;vdb=variation;vf=12971171) | ring finger protein 144B , lbrdc2 | E3 ubiquitin ligase |
| rs29225085 | 47229758 | B6 | intron variant | [00000038068](https://uswest.ensembl.org/Mus_musculus/Gene/Variation_Gene/Table?db=core;g=ENSMUSG00000038068;source=dbSNP;v=rs29225085;vdb=variation;vf=12974611) | ring finger protein 144B , lbrdc2 | E3 ubiquitin ligase |
| rs29914889 | 47764476 | B6 | intergenic variant |  |  |  |
| rs6244558 | 47815991 | B6 | intergenic variant |  |  |  |
| rs220959940 | 48010713 | B6 | intron variant | [00000047324](https://uswest.ensembl.org/Mus_musculus/Gene/Variation_Gene/Table?db=core;g=ENSMUSG00000047324;source=dbSNP;v=rs220959940;vdb=variation;vf=13000274) | RIKEN cDNA 4931429P17 |  |
| rs29568118 | 48119885 | B6 | intron variant | [00000097622](https://uswest.ensembl.org/Mus_musculus/Gene/Variation_Gene/Table?db=core;g=ENSMUSG00000097622;source=dbSNP;v=rs29568118;vdb=variation;vf=13003397) | RIKEN cDNA A330033J07 |  |
| rs30191571 | 48607862 | B6 | intron variant | [00000038042](https://uswest.ensembl.org/Mus_musculus/Gene/Variation_Gene/Table?db=core;g=ENSMUSG00000038042;source=dbSNP;v=rs30191571;vdb=variation;vf=13016926) | protein tyrosine phosphatase domain containing 1 |  |
|  | **49340961** |  |  | [**00000037966**](https://uswest.ensembl.org/Mus_musculus/Gene/Summary?db=core;g=ENSMUSG00000037966;r=13:1-100000000) | **Ninjurin 1 (NINJ1)** | Terminal Pore Regulation |
|  | **49356426** |  |  | **1110007C09Rik** | **CARD19** |  |
| rs37780795 | 49403959 | B6 | intergenic variant |  |  |  |
| rs6330796 | 51046190 | B6 | intergenic variant |  |  |  |
| rs226625541 | 51769402 | 129 | intron variant | 00000021451 | Sema4d | Signaling |
| rs46751182 | 52014198 | B6 |  | 00000793968 |  |  |
| rs30070966 | 52018425 | 129 | downstream gene variant | [00000102173](https://uswest.ensembl.org/Mus_musculus/Gene/Variation_Gene/Table?db=core;g=ENSMUSG00000102173;source=dbSNP;v=rs30070966;vdb=variation;vf=2819030) | predicted gene |  |
| rs29231157 | 52019172 | 129 | downstream gene variant | [00000102173](https://uswest.ensembl.org/Mus_musculus/Gene/Variation_Gene/Table?db=core;g=ENSMUSG00000102173;source=dbSNP;v=rs29231157;vdb=variation;vf=2007024) | predicted gene |  |
| rs36690691 | 52951172 | 129 | intergenic variant |  |  |  |
| rs29529592 | 53233552 | 129 | intron variant | [00000021464](https://uswest.ensembl.org/Mus_musculus/Gene/Variation_Gene/Table?db=core;g=ENSMUSG00000021464;source=dbSNP;v=rs29529592;vdb=variation;vf=2294845) | ntrk2 |  |
| rs49400466 | 53412842 | 129 | intron variant | [00000107008](https://uswest.ensembl.org/Mus_musculus/Gene/Variation_Gene/Table?db=core;g=ENSMUSG00000107008;source=dbSNP;v=rs49400466;vdb=variation;vf=11263192) | predicted gene |  |
| rs30058409 | 53813956 | 129 | intergenic variant |  |  |  |
| rs29551959 | 54964992 | 129 | intron variant | [00000025876](https://uswest.ensembl.org/Mus_musculus/Gene/Variation_Gene/Table?db=core;g=ENSMUSG00000025876;source=dbSNP;v=rs29551959;vdb=variation;vf=2316372) | unc5h1 |  |
| rs30004717 | 54967613 | 129 | intron variant | [00000025876](https://uswest.ensembl.org/Mus_musculus/Gene/Variation_Gene/Table?db=core;g=ENSMUSG00000025876;source=dbSNP;v=rs30004717;vdb=variation;vf=2754993) | unc5h1 |  |
| rs29927068 | 55063173 | 129 | intron variant | [00000025878](https://uswest.ensembl.org/Mus_musculus/Gene/Variation_Gene/Table?db=core;g=ENSMUSG00000025878;source=dbSNP;v=rs29927068;vdb=variation;vf=2679785) | ubiquitin interaction motif containing 1 |  |
| rs29239941 | 56024612 | 129 | intron variant | [00000114493](https://uswest.ensembl.org/Mus_musculus/Gene/Variation_Gene/Table?db=core;g=ENSMUSG00000114493;source=dbSNP;v=rs29239941;vdb=variation;vf=2015597) | predicted gene |  |
| rs29234727 | 56057642 | 129 | intron variant | [00000114493](https://uswest.ensembl.org/Mus_musculus/Gene/Variation_Gene/Table?db=core;g=ENSMUSG00000114493;source=dbSNP;v=rs29234727;vdb=variation;vf=2010537) | predicted gene |  |
| rs29227915 | 56074012 | 129 | 3' UTR variant | [00000015937](https://uswest.ensembl.org/Mus_musculus/Gene/Variation_Gene/Table?db=core;g=ENSMUSG00000015937;source=dbSNP;v=rs29227915;vdb=variation;vf=2003879) | macroH2A.1 histone |  |
| rs46196633 | 56077106 | 129 | intron variant | [00000015937](https://uswest.ensembl.org/Mus_musculus/Gene/Variation_Gene/Table?db=core;g=ENSMUSG00000015937;source=dbSNP;v=rs46196633;vdb=variation;vf=8428273) | macroH2A.1 histone |  |
| rs3720782 | 56229525 | 129 | intron variant | [00000097361](https://uswest.ensembl.org/Mus_musculus/Gene/Variation_Gene/Table?db=core;g=ENSMUSG00000097361;source=dbSNP;v=rs3720782;vdb=variation;vf=99032) | RIKEN cDNA 4930550C17 gene |  |
| rs3700819 | 57253190 | B6 | intergenic variant |  |  |  |
| rs30059311 | 57271612 | B6 | intergenic variant |  |  |  |
| rs29249300 | 57408045 | B6 | intergenic variant |  |  |  |
| rs30078817 | 57485175 | B6 | intron variant | [00000056222](https://uswest.ensembl.org/Mus_musculus/Gene/Variation_Gene/Table?db=core;g=ENSMUSG00000056222;source=dbSNP;v=rs30078817;vdb=variation;vf=13294976) | Ticn1/Spock1 | Cell-cell interactions |
| rs257680153 | 58007116 | B6 | intron variant | [00000014164](https://uswest.ensembl.org/Mus_musculus/Gene/Variation_Gene/Table?db=core;g=ENSMUSG00000014164;source=dbSNP;v=rs257680153;vdb=variation;vf=13314341) | klhl3 | Nephron ion transport |
| rs13481832 | 58837796 | B6 | splice region variant | [00000055254](https://uswest.ensembl.org/Mus_musculus/Gene/Variation_Gene/Table?db=core;g=ENSMUSG00000055254;source=dbSNP;v=rs13481832;vdb=variation;vf=13343595) | neurotrophic tyrosine kinase, receptor, type 2 | Neuronal homeostasis and development |
| rs30250735 | 59737415 | B6 | downstream gene variant | [00000181528](https://uswest.ensembl.org/Mus_musculus/Transcript/Summary?db=core;g=ENSMUSG00000097502;r=13:59881444-59886999;t=ENSMUST00000181528.2;v=rs30250735;vdb=variation;vf=13376264) | RIKEN cDNA 4930528D03 |  |

SNPs from DartMouse SNP genetic background check 10 megabases upstream and downstream of the *Ninj1/Card19* chromosome 13 locus. SNP identification and chromosomal position are indicated. SNPs are indicated as wildtype (B6) or 129SvEvBrd (129). The change, gene ID, gene name, and known function are listed. *Ninj1* and *Card19* are listed for reference.
