# Supplementary material for: Genetic targeting of Card19 is linked to disrupted NINJ1 expression, impaired cell lysis, and increased susceptibility to Yersinia infection
Source: PLoS Pathog. 2021 Oct 14;17(10):e1009967. doi: 10.1371/journal.ppat.1009967 (PMC8547626; doi:10.1371/journal.ppat.1009967)
Supplement: S3 Table — Chromosome 13 results are displayed from whole exome sequencing of Card19lxcn mice. (DOCX) [file ppat.1009967.s009.docx]

**S3 Table: Chromosome 13 Whole Exome Sequencing Results**

| **Gene ID** | **Gene Name** | **Position** | **Change** | **Function and notes** |
| --- | --- | --- | --- | --- |
| Spata31 | spermatogenesis associated 31 | 13, 34.21 | R645 to stop | spermatogenesis; testis specific |
| Nlrp4f | NLR family, pyrin domain containing 4F | 13, 34.45 | E604Q | organelle development |
| Gm10324 | predicted gene 10324 | 13, 34.51 | S310C, Y316H, A363V, K458R, F551Y | testis, placenta specific |
| 2410141K09Rik | RIKEN cDNA 2410141K09 gene | 13, 34.52 | several intergenic region Indels | testis specific |
| 2410141K09Rik | RIKEN cDNA 2410141K09 gene | 13, 34.52 | intron variant, 153A>G | testis specific |
| Adcy2 | adenylate cyclase 2 | 13, 35.55 | intergenic region, 6802150A>G | cAMP signaling |
| Cmya5 | cardiomyopathy associated 5 | 13, 47.81 | A3414P | anchoring protein for PKA, skeletal muscle regeneration |
| **Card19** |  | **13, 49.35-36** |  |  |
| Naip1 | NLR family, apoptosis inhibitory protein 1 | 13, 53.18 | W288L | inhibits apoptosis |
| Tmem267 | transmembrane protein 267 | 13, 67.25 | intron variant, 1131C>T | testis specific |

Chromosome 13 results are displayed from whole exome sequencing of *Card19^lxcn^* mice.
