# Supplementary material for: Genetic targeting of Card19 is linked to disrupted NINJ1 expression, impaired cell lysis, and increased susceptibility to Yersinia infection
Source: PLoS Pathog. 2021 Oct 14;17(10):e1009967. doi: 10.1371/journal.ppat.1009967 (PMC8547626; doi:10.1371/journal.ppat.1009967)
Supplement: S4 Table — RNA-Seq results from untreated B6 and Card19lxcn BMDMs. (DOCX) [file ppat.1009967.s010.docx]

**S4 Table: RNA-Seq Results: Untreated B6 BMDMs vs. Untreated *Card19^lxcn^* BMDMs**

| **Gene ID** | **B6 Average** | **Card19^lxcn^ Average** | **Log Fold Change** | **Function** |
| --- | --- | --- | --- | --- |
| Sirt5 | 0.44 | -6.27 | -6.71 | NAD+ regulation |
| Ninj1 | 6.45 | 4.92 | -1.53 | Plasma membrane rupture regulation |
| Axl | 6.59 | 5.51 | -1.07 | Inhibits TLR signaling |
| Wdfy1 | 3.82 | 4.83 | 1 | Positively regulates TLR signaling |
| Cxcl14 | 5.47 | 7.14 | 1.67 | Chemokine for innate immune cells |

RNA-Seq results from untreated B6 and *Card19^lxcn^* BMDMs.
