# Supplementary material for: Genetic targeting of Card19 is linked to disrupted NINJ1 expression, impaired cell lysis, and increased susceptibility to Yersinia infection
Source: PLoS Pathog. 2021 Oct 14;17(10):e1009967. doi: 10.1371/journal.ppat.1009967 (PMC8547626; doi:10.1371/journal.ppat.1009967)
Supplement: S5 Table — Reagents and resources utilized are listed in S5 Table. (DOCX) [file ppat.1009967.s011.docx]

**S5 Table. Key resources and reagents.**

| **REAGENT or RESOURCE** | **SOURCE** | **IDENTIFIER** |  |
| --- | --- | --- | --- |
| **Antibodies** | | | |
| Anti-CARD19 polyclonal antibody | Atlas Antibodies | Cat# HPA010990, RRID:AB_2668400 |  |
| Caspase-1 antibody | Genetech | N/A |  |
| Rabbit polyclonal to Caspase-3 | Cell Signaling Technologies | Cat# 9662S, RRID:AB_10694681 |  |
| Rat Anti-Mouse Caspase-8 Monoclonal Antibody, Clone 1G12 | Enzo Life Technologies | Cat# ALX-804-447-C100, RRID:AB_2050952 |  |
| Anti-DFNA5/GSDME antibody [EPR19859] – N-terminal | Abcam | Cat# ab215191,  RRID:AB_2737000 |  |
| Rabbit monoclonal [EPR19828] to GSDMD | Abcam | Cat# ab209845 |  |
| Rabbit Polyclonal to HDAC1 | Cell Signaling Technologies | Cat# 2062, RRID:AB_2118523 |  |
| HA-Tag (C29F4) Rabbit mAb | Cell Signaling | Cat#3724T |  |
| HMGB1 antibody - ChIP Grade | Abcam | Cat# ab18256, RRID:AB_444360 |  |
| Purified Mouse anti-Ninjurin GNE 425 | Genetech | N/A |  |
| Monoclonal Anti-alpha-Tubulin antibody produced in mouse | Sigma-Aldrich | Cat# T5168, RRID:AB_477579 |  |
| Goat Anti-Rabbit IgG (H+L) Antibody, Alexa Fluor 488 Conjugated | Molecular Probes | A-11008, RRID:AB_143165 |  |
| Goat anti-Mouse IgG (H+L) Cross-Adsorbed Secondary Antibody, Alexa Fluor 514 | Thermo Fisher Scientific | Cat# A-31555, RRID:AB_2536171 |  |
| Alexa Fluor® 647 Phalloidin antibody | Thermo Fisher Scientific | Cat# A22287, RRID:AB_2620155 |  |
| Mouse Anti-beta-Actin Monoclonal Antibody, Unconjugated, Clone AC-74 | Sigma-Aldrich | Cat# A2228, RRID:AB_476697 |  |
| Peroxidase-AffiniPure Goat Anti-Rat IgG (H+L) (min X Hu,Bov,Hrs,Rb Sr Prot) antibody | Jackson Labs | Cat# 112-035-143, RRID:AB_2338138 |  |
| Peroxidase-AffiniPure Goat Anti-Rabbit IgG (H+L) (min X Hu,Ms,Rat Sr Prot) antibody | Jackson Labs | Cat# 111-035-144, RRID:AB_2307391 |  |
| Anti-mouse IgG, HRP-linked Antibody | Cell Signaling Technologies | Cat# 7076, RRID:AB_330924 |  |
| IL-1a antibody | BD Biosciences | Cat# 550604, RRID:AB_393776 |  |
| Biotin anti-mouse IL-1a antibody | BioLegend | Cat# 512504, RRID:AB_2124220 |  |
| Anti-Mouse/Rat IL-1 beta Purified 500 ug antibody | Thermo Fisher Scientific | Cat# 14-7012-85, RRID:AB_468397 |  |
| Anti-Mouse IL-1 beta Biotin 500 ug antibody | Thermo Fisher Scientific | Cat# 13-7112-85, RRID:AB_466925 |  |
| Rat Anti-IL-6 Monoclonal Antibody, Unconjugated, Clone MP5-20F3 | BD Biosciences | Cat# 554400, RRID:AB_398549 |  |
| Rat Anti-IL-6 Monoclonal Antibody, Biotin Conjugated, Clone MP5-32C11 | BD Biosciences | Cat# 554402, RRID:AB_395368 |  |
| Rat Anti-IL-12 (p40 / p70) Monoclonal Antibody, Unconjugated, Clone C15.6 | BD Biosciences | Cat# 551219, RRID:AB_394097 |  |
| Rat Anti-IL-12 (p40 / p70) Monoclonal Antibody, Biotin Conjugated, Clone C17.8 | BD Biosciences | Cat# 554476, RRID:AB_395419 |  |
| IFN gamma Monoclonal Antibody (AN-18), eBioscience(TM) | Thermo Fisher Scientific | Cat# 14-7313-85, RRID:AB_468472 |  |
| IFN gamma Monoclonal Antibody (R4-6A2), Biotin, eBioscience(TM) | Thermo Fisher Scientific | Cat# 13-7312-85, RRID:AB_466939 |  |
| Goat anti-Mouse IgG1 Secondary Antibody, Alexa Fluor 488 conjugate | Thermo Fisher Scientific | Cat# A-21121, RRID:AB_2535764 |  |
| Goat anti-Mouse IgG1 Cross-Adsorbed Secondary Antibody, Alexa Fluor 647 | Thermo Fisher Scientific | Cat# A-21240, RRID:AB_2535809 |  |
| Goat anti-Mouse IgG2a Cross-Adsorbed Secondary Antibody, Alexa Fluor 488 | Thermo Fisher Scientific | Cat# A-21131, RRID:AB_2535771 |  |
| Goat anti-Rabbit IgG (H+L) Highly Cross-Adsorbed Secondary Antibody, Alexa Fluor 647 | Thermo Fisher Scientific | Cat# A-21245, RRID:AB_2535813 |  |
| Goat anti-Rabbit IgG (H+L) Secondary Antibody, Alexa Fluor 546 | Thermo Fisher Scientific | Cat# A-11010,  RRID: AB_143156 |  |
| Goat anti-Mouse IgG (H+L) Secondary Antibody, Alexa Fluor 546 | Thermo Fisher Scientific | Cat# A-11003,  RRID: AB_2534071 |  |
| **Bacterial and Virus Strains** | | | |
| *Yersinia pseudotuberculosis* IP2777 | Jim Bliska | [1,2] |  |
| *Yersinia pseudotuberculosis* IP2666 | Jim Bliska | [1,3] |  |
| *Yersinia pseudotuberculosis* IP2666 ΔyopEJK | Erin Zwack, Igor Brodksy | [4] |  |
| *Salmonella* Typhimurium SL1344 | [5] |  |  |
| *ΔoatA Staphylococcus aureus* | Jonathan Kagan | [6] |  |
| *E. coli* DH5α | N/A |  |  |
| ER-HoxB8 Virus | David Sykes | [7] |  |
| **Biological Samples** |  |  |  |
| *Gsdmd^-/-^* Bone Marrow | Russell Vance | [8] |  |
| *Mavs^-/-^* Bone Marrow | Carolina Lopez, Jackson Labs | Cat# 008634 |  |
| *Casp11*^-/-^ Bone Marrow | Junying Yuan | [9] |  |
| *Sarm1(AGS3)^-/-^*, *Sarm1(AGS12)^-/-^*, *Sarm1(AD)^‑/-^* Bone Marrow | Adolfo Garcia-Sastre | [10] |  |
| *Ninj1^-/-^ iBMDMs* | Vishva Dixit | [11] |  |
| **Chemicals, Peptides, and Recombinant Proteins** | | | |
| Hoechst | Thermo Fisher Scientific | Cat# 62249 |  |
| Fluormount G | Thermo Fisher Scientific | Cat# 00-4958-02 |  |
| Lipopolysaccharide from E Coli | Sigma-Aldrich | Cat# L2880 |  |
| Pam3CSK4 | Invivogen | Cat# tlrl-pms |  |
| Cycloheximide from microbial | Sigma-Aldrich | Cat# C7698 |  |
| zVAD(Ome)-FMK | SM Biochemicals | Cat# SMFMK001 |  |
| z-IETD-FMK | SM Biochemicals | Cat# SMFMK004 |  |
| Adenosine 5ʹ-Triphosphate, Disodium Salt | Millipore | Cat# 1191 |  |
| Gentamicin Sulfate | Sigma-Aldrich | Cat# G1914 |  |
| Irgasan | Sigma-Aldrich | Cat# 72779 |  |
| Streptamycin Sulfate | Gold Technologies | Cat# G-400-1 |  |
| Staurosporine from *Streptomyces sp.* | Sigma-Aldrich | Cat# S5921 |  |
| Necrostatin (Nec-1) | EMD Chemicals | Cat# 480065 |  |
| MitoTracker CMXRos | Life Technologies | Cat# M7512 |  |
| Propidium iodide | Thermo Fisher Scientific | Cat# P3566 |  |
| ECL Western Blotting Substrate | Thermo Fisher Scientific | Cat# 32106 |  |
| SuperSignal West Femto Maximum Sensitivity Substrate | Thermo Fisher Scientific | Cat# 34095 |  |
| Complete, Mini, EDTA-Free Protease Inhibitor Cocktail | Roche | Cat# 11836170001 |  |
| Streptavidin HRP | BD Biosciences | Cat# 554066 |  |
| Recombinant Mouse TNF-alpha | BioLegend | Cat# 575206 |  |
| Recombinant Mouse IFN-gamma protein | eBioscience | Cat# 485-MI-100 |  |
| Mouse IL-1beta Recombinant Protein | R&D | Cat# 14-8012-80 |  |
| Recombinant IL-6 Standard | R&D | Cat# 406-ML-005 |  |
| Recombinant Mouse IL-12 protein | R&D | Cat# 419-ML-010 |  |
| Recombinant Mouse IL-1 alpha | R&D | Cat# 400-ML-005 |  |
| Lipofectamine 2000 | Invitrogen | Cat# 11668027 |  |
| 16% Paraformaldehyde Aqueous Solution, EM Grad | Electron Microscopy Services | Cat# 15710 |  |
| Mounting Media, PPD in 90% Glycerol | [12] |  |  |
| DAPI | Molecular Probes | Cat# D-1306 |  |
| Todd Hewitt Broth for microbiology | Sigma-Aldrich | Cat# T1438 |  |
| Dextran Dye-150 | Millipore Sigma | Cat# FD150S |  |
| **Commercial Assays** | | | |
| Plasma Membrane Protein Extraction Kit | Abcam | Cat# ab65400 |  |
| LDH Cytotoxicity Detection Kit | Takara Bio | Cat# MK401 |  |
| CellTiter Glo Luminescent Cell Viability Assay | Promega | Cat# G7571 |  |
| Plasmid Maxiprep Kit | Qiagen | Cat# 12162 |  |
| Caspase-Glo 8 Assay | Fisher Scientific | Cat# PRG8201 |  |
| **Experimental Models: Cell Lines** | | | |
| HEK 293T Cells | ATCC |  |  |
| **Experimental Models: Organisms/Strains** | | | |
| *Card19^lxcn^* Mice | Brian Schaefer | [13] |  |
| *Card19^ΔCARD^* Mice | This paper |  |  |
| *Card19^Null^* Mice | This paper |  |  |
| C57BL/6J Mice (B6) | Jackson Labs | Cat# 000664 |  |
| *Casp1*/*Casp11*^-/-^ Mice | Jackson Labs | Cat# 016621 |  |
| *Ripk3/Casp8^-/-^* Mice | Doug Green | [14] |  |
| *Sarm1(MSD)^-/-^* | Adriano Aguzzi | [15] |  |
| *Ripk3*^-/-^ Mice | Kim Newton, Vishva Dixit | [16] |  |
| **Oligonucleotides** | | | |
| *Card19^lxcn^* Wt genotyping primers, (297 bp) CATGGATGTACAGAGCTCGGTAA, CGTTGCCCTGGAGACACAGTATT | IDT | This paper |  |
| *Card19^lxcn^* Knockout genotyping primers (281 bp)  CGGAATTGATCCCGCTCGAA, CGTTGCCCTGGAGACACAGTATT | IDT | This paper |  |
| *Card19^ΔCARD^* Sequencing Primer (250 bp)  CTTGGGAAAAGTGTGGCTTTTGT, TCCTCCAGTCTGTCCATGTGGGGATTTT | Sigma | This paper |  |
| *Card19^Null^* Sequencing primer (298 bp)  TCGGTTTCTTCATCCAGGAG,  GAGGCAGCCACTGGGTATAA | Sigma | This paper |  |
| **Recombinant DNA** | | | |
| pcDNA3.1+/CARD19-FLAG | GenScript | Cat# OMu021914D |  |
| pcDNA3.1+ | Igor Brodsky |  |  |
| pMSCV2.2 | Igor Brodsky |  |  |
| pMSCV2.2/CARD19 | This paper |  |  |
| pCL-Eco | Igor Brodsky |  |  |
| mNINJ1/BH1.11 | Vishva Dixit | [11] |  |
| BH1.11 | Vishva Dixit | [11] |  |
| pBO | Vishva Dixit | [11] |  |
| **Software and Algorithms** | | | |
| FIJI | [17] | https://imagej.net/Fiji/Downloads |  |
| Volocity 6.3 | PerkinElmer | http://cellularimaging.perkinelmer.com/downloads/detail.php?id=14 |  |
| Prism 5.0 | GraphPad | https://www.graphpad.com/scientific-software/prism/ |  |
| R version 4.0.3 | R | https://www.r-project.org/ |  |
| RStudio version 1.2.5042 | RStudio | https://rstudio.com/ |  |
| Other | | | |

1. Simonet M, Falkow S. Invasin expression in Yersinia pseudotuberculosis. Infection and immunity. 1992;60(10):4414-7. Epub 1992/10/01. PubMed PMID: 1398952; PubMed Central PMCID: PMC257481.

2. McPhee JB, Mena P, Zhang Y, Bliska JB. Interleukin-10 induction is an important virulence function of the Yersinia pseudotuberculosis type III effector YopM. Infection and immunity. 2012;80(7):2519-27. Epub 2012/05/02. doi: 10.1128/IAI.06364-11

10.1128/IAI.06364-11. Epub 2012 Apr 30. PubMed PMID: 22547545; PubMed Central PMCID: PMC3416464.

3. Grabenstein Jens P, Marceau M, Pujol C, Simonet M, Bliska James B. The Response Regulator PhoP of Yersinia pseudotuberculosis Is Important for Replication in Macrophages and for Virulence. Infection and immunity. 2004;72(9):4973-84. doi: 10.1128/IAI.72.9.4973-4984.2004.

4. Zwack EE, Snyder AG, Wynosky-Dolfi MA, Ruthel G, Philip NH, Marketon MM, et al. Inflammasome Activation in Response to the Yersinia Type III Secretion System Requires Hyperinjection of Translocon Proteins YopB and YopD. mBio. 2015;6(1):e02095-14. doi: 10.1128/mBio.02095-14. PubMed PMID: PMC4337566.

5. Hoiseth SK, Stocker BA. Aromatic-dependent Salmonella typhimurium are non-virulent and effective as live vaccines. Nature. 1981;291(5812):238-9. Epub 1981/05/21. PubMed PMID: 7015147.

6. Evavold CL, Ruan J, Tan Y, Xia S, Wu H, Kagan JC. The Pore-Forming Protein Gasdermin D Regulates Interleukin-1 Secretion from Living Macrophages. Immunity. 2018;48(1):35-44 e6. doi: 10.1016/j.immuni.2017.11.013

10.1016/j.immuni.2017.11.013. Epub 2017 Nov 28. PubMed PMID: 29195811; PubMed Central PMCID: PMCPMC5773350.

7. Wang GG, Calvo KR, Pasillas MP, Sykes DB, Häcker H, Kamps MP. Quantitative production of macrophages or neutrophils ex vivo using conditional Hoxb8. Nature Methods. 2006;3(4):287-93. doi: 10.1038/nmeth865.

8. Rauch I, Deets KA, Ji DX, von Moltke J, Tenthorey JL, Lee AY, et al. NAIP-NLRC4 Inflammasomes Coordinate Intestinal Epithelial Cell Expulsion with Eicosanoid and IL-18 Release via Activation of Caspase-1 and -8. Immunity. 2017;46(4):649-59. doi: 10.1016/j.immuni.2017.03.016.

9. Wang S, Miura M, Jung Y-k, Zhu H, Li E, Yuan J. Murine Caspase-11, an ICE-Interacting Protease, Is Essential for the Activation of ICE. Cell. 1998;92(4):501-9. doi: <https://doi.org/10.1016/S0092-8674(00)80943-5>.

10. Uccellini MB, Bardina SV, Sánchez-Aparicio MT, White KM, Hou Y-J, Lim JK, et al. Passenger Mutations Confound Phenotypes of SARM1-Deficient Mice. Cell reports. 2020;31(1):107498. doi: 10.1016/j.celrep.2020.03.062. PubMed PMID: 32268088.

11. Kayagaki N, Kornfeld OS, Lee BL, Stowe IB, O’Rourke K, Li Q, et al. NINJ1 mediates plasma membrane rupture during lytic cell death. Nature. 2021. doi: 10.1038/s41586-021-03218-7.

12. Traver MK, Paul S, Schaefer BC. T Cell Receptor Activation of NF-κB in Effector T Cells: Visualizing Signaling Events Within and Beyond the Cytoplasmic Domain of the Immunological Synapse. In: Baldari CT, Dustin ML, editors. The Immune Synapse: Methods and Protocols. New York, NY: Springer New York; 2017. p. 101-27.

13. Rios KE, Kashyap AK, Maynard SK, Washington M, Paul S, Schaefer BC. CARD19, the protein formerly known as BinCARD, is a mitochondrial protein that does not regulate Bcl10-dependent NF-κB activation after TCR engagement. Cellular Immunology. 2020;356:104179. doi: <https://doi.org/10.1016/j.cellimm.2020.104179>.

14. Oberst A, Dillon CP, Weinlich R, McCormick LL, Fitzgerald P, Pop C, et al. Catalytic activity of the caspase-8-FLIP(L) complex inhibits RIPK3-dependent necrosis. Nature. 2011;471(7338):363-7. Epub 2011/03/04. doi: 10.1038/nature09852

10.1038/nature09852. Epub 2011 Mar 2. PubMed PMID: 21368763; PubMed Central PMCID: PMC3077893.

15. Zhu C, Li B, Frontzek K, Liu Y, Aguzzi A. SARM1 deficiency up-regulates XAF1, promotes neuronal apoptosis, and accelerates prion disease. J Exp Med. 2019;216(4):743-56. Epub 2019/03/08. doi: 10.1084/jem.20171885. PubMed PMID: 30842236; PubMed Central PMCID: PMCPMC6446871.

16. Newton K, Sun X, Dixit VM. Kinase RIP3 is dispensable for normal NF-kappa Bs, signaling by the B-cell and T-cell receptors, tumor necrosis factor receptor 1, and Toll-like receptors 2 and 4. Mol Cell Biol. 2004;24(4):1464-9. PubMed PMID: 14749364; PubMed Central PMCID: PMCPMC344190.

17. Schindelin J, Arganda-Carreras I, Frise E, Kaynig V, Longair M, Pietzsch T, et al. Fiji: an open-source platform for biological-image analysis. Nature Methods. 2012;9:676. doi: 10.1038/nmeth.2019 <https://www.nature.com/articles/nmeth.2019#supplementary-information>.
